# Healthy Lifestyle Behaviors of Research Assistants and Final-Year Students in the Medical Faculty

**DOI:** 10.5152/eurasianjmed.2024.24412

**Published:** 2024-10-01

**Authors:** Nurdan Torun, Aysun Aras, Serhat Vançelik, Zahide Koşan, Sinan Yilmaz

**Affiliations:** Department of Public Health, Atatürk University Faculty of Medicine, Erzurum, Türkiye

**Keywords:** Healthy lifestyle, physician, university

## Abstract

**Background:**

This study aimed to identify the health-related behaviors and associated factors among research assistants and final-year students at a Faculty of Medicine.

**Methods:**

In this cross-sectional study, a total of 712 assistant physicians and final-year medical students participated. Data collection involved using a survey form to inquire about participants’ sociodemographic characteristics and the “Healthy Lifestyle Behavior Scale II (HPLP II).” Descriptive statistics, *t*-test, ANOVA, Kruskal–Wallis, and Mann–Whitney *U* tests with Bonferroni correction were employed for data analysis.

**Results:**

Among the participants, 52.5% were assistant physicians, 47.5% were final-year students, and 50.4% were women. Physicians scored an average of 120.0 ± 16.5 on the HPLP II, with the lowest in the physical activity subscale and the highest score in the spiritual development subscale. Healthy lifestyles were more prevalent in those aged 23-28, in those not married and who are childless, women, non-shift workers, non-smokers, those with chronic conditions, and those perceiving their health as good or fair.

**Conclusion:**

Factors influencing healthy lifestyle behaviors include age, marital status, parenthood, smoking and alcohol habits, work unit, health perception, and chronic diseases. Analyzing and conducting studies on modifiable characteristics are recommended to enhance health.

**Main Points**

- People should have more time for themselves to practice stress management techniques.
- Participants with poor health perception had lower interpersonal relationships subscale scores.
- Participants who smoked cigarettes had lower overall scale scores.

## Introduction

The basic condition for people’s desire to live a quality, successful, and peaceful life is to live healthily and grow old. In the past, health has been defined only as the absence of disability or disease. However, this definition only emphasizes the concept of disease, and anyone without complaints or symptoms is considered healthy.^1^ The most widely accepted definition of health today is that of the World Health Organization. It defines health as “not only the absence of disease or infirmity, but also a state of complete physical, mental, and social well-being.”^2^

Interventions to increase and ensure individuals’ control over their health status are defined as health promotion. The primary goal of health promotion is to communicate healthy behaviors to communities and to ensure that they are adopted by communities. In this context, it can be said that health promotion interventions are the process of increasing the health responsibility of individuals. Health promotion is also a process of helping individuals make voluntary decisions to maximize their mental and physical health and improve the environment in which they live.^3^

Health promotion is not only an attempt to improve individuals’ personal skills and abilities but also a social and political concept to improve economic, environmental, and social conditions. Community involvement, empowerment, and joint efforts are important concepts in health promotion as they enable individuals to have more effective control over decisions affecting their health.^4^

Health is a fundamental human right, and the promotion, protection, and maintenance of health are the chief targets of health policies and the responsibility of each individual.^3^ Healthy lifestyle behaviors involve individuals controlling all their health-related behaviors and choosing actions to increase their state of health while performing their life activities.^4^ People who adopt healthy lifestyles can maintain and improve their wellbeing.^5^

Healthcare workers should first organize their own lifestyles and make them healthy, given their professional obligations and their role in society.^6^ In a study conducted in America, it has been found that it is more difficult for physicians to give advice to the patients they care for if they themselves do not follow the advice they give.^7^

Healthcare workers should adopt healthy lifestyles to protect and improve their own health, recognizing that they have important roles and responsibilities in protecting and improving the health of society.

Studies on healthy lifestyle behaviors in Türkiye and abroad have generally been conducted on similar populations. Studies on physicians and physician candidates are very few. The aim of our study is to assess the healthy lifestyle behaviors of resident and final-year doctors at Atatürk University and to disclose the related factors. It is anticipated that the results of the research will be a contribution to the literature on these issues.

## Material and Methods

This study was cross-sectional and consisted of 423 resident physicians who specialized in a university hospital between 2021 and 2022 and 375 final-year medical students. No sample selection was made, and all of the population was attempted to be reached. Eighty-eight percent of the resident physicians (374 people) and ninety percent of the final-year medical students (338 people) were reached. The study was performed between January 2022 and June 2022 under observation. The questionnaire used for data collection consisted of the “Personal Information Form” and the “Healthy Lifestyle Behaviors Scale II.” This study was approved by Ethics Committee of Atatürk University Clinical Research —(Approval No. 37, Date: 27.01.2022). Written informed consent was obtained from all participants who participated in this study.

The Health Promoting Lifestyle Profile Scale II (HPLP II) was initially developed by Walker et al. in 19 87^8^ and underwent revisions in 1996.^9^ In our investigation, we utilized the second version, which had its validity and reliability assessments conducted by Bahar et al. The HPLP II comprises 52 items categorized into 6 sub-dimensions: spiritual development, health responsibility, physical activity, diet, interpersonal relationships, and stress management.

Employing a 4-point Likert-type scale, respondents assigned scores of 1 for “never,” 2 for “sometimes,” 3 for “frequently,” and 4 for “regularly” to each item. The cumulative score of the scale serves as an indicator of healthy lifestyle behaviors. All items on the scale are positively framed. Scores on the scale can range from a minimum of 52 to a maximum of 208. An increase in scores reflects a higher level of expressed health behaviors by individuals.^10^

The data of the study were analyzed using descriptive statistics, ANOVA, *t*-test, Kruskal–Wallis, and Mann–Whitney *U* analyses with Bonferroni correction.

## Results

In our research, 52.5% (n = 374) of the participants consisted of resident physicians, while 47.5% (n = 338) were final-year medical students. Among the physicians, 50.4% were identified as female (n = 359), and 49.6% were male (n = 353). The average age was 26.6 ± 3.4 years, ranging from 23 to 46. Three-quarters of the participants (75%, n = 534) fell within the age range of 23-28, with 22.5% (n = 160) aged between 29-34, and only 2.5% (n = 18) aged 35 and older. Concerning marital status, 22.8% (n = 162) were married, while 77.2% (n = 560) were unmarried. In terms of parenthood, 12.1% (n = 86) of the physicians had children, whereas 87.9% (n = 626) did not. 18.7% (n = 133), 63.8% (n = 454), and 17.6% (n = 125) of doctors reported that their income was good, medium, and poor, respectively (Table 1).

In terms of smoking status, 20.9% (n = 149) of doctors were smokers, 75.7% (n = 539) were non-smokers, and 3.4% (n = 24) reported quitting. 90.2% (n = 642) of the participants did not have a chronic illness. 9.8% (n = 70) of the physicians were taking at least one medication continuously. Approximately 62.1% (n = 442) of the participants rated their health status as good, 34.7% (n = 247) as moderate, and 3.2% (n = 23) as poor.

### Healthy Lifestyle Behaviors and Associated Factors

Score of the participants in the HPLP II was found to be 120.0 ± 16.5. When the subscale scores of assistants and final-year medical students were examined, the interpersonal relations score was 24.1 ± 3.9, the mean score of the nutrition sub-dimension was 18.9 ± 3.6, the physical activity score was 16.0 ± 4.7, the health responsibility score was 18.7 ± 3.9, the spiritual development score was 24.8 ± 4.4, and the stress management score was 17.8 ± 3.5. It was observed that the participants received the lowest score from the physical activity sub-dimension and the highest score from the spiritual development sub-dimension. In our study, the scores for the interpersonal relationships sub-dimension and the health responsibility sub-dimension were significantly higher for women (*P* < .05).

In relation to the smoking habits of physicians, both the overall scale and the health responsibility sub-dimension scores exhibited a notable increase in favor of non-smokers (*P* < .05). Stress management scores were found to be significantly higher in those without children, stress management and physical activity scores in singles, and stress management scores in the 23-28 age group (*P* < .05)(Table 2). Analyzing physicians’ chronic disease status (Table 2), the health responsibility sub-dimension score was notably elevated in those with chronic diseases (*P* < .05).

Noteworthy differences emerged specifically in the interpersonal relationships and physical activity sub-dimensions based on physicians’ self-rated health (*P *< .05). In subsequent pairwise comparisons, disparities in the interpersonal relations sub-dimension were identified between those with good and poor health, while significance in the physical activity sub-dimension was evident between groups with good-poor and poor-moderate health (*P* < .05). Participants who perceived their health as good (Table 2) demonstrated significantly higher scores in interpersonal relationships compared to those with poor health. Conversely, participants who rated their health as poor exhibited notably higher scores in the physical activity sub-dimension compared to others (*P* < .05). When the healthy lifestyle behaviors of assistant and intern Physicians were compared, the general score, stress management and physical activity scores were found to be significantly higher in intern Physicians (*P* < .05) (Table 3). 

## Discussion

In our study, the scores for the interpersonal relationships sub-dimension and the health responsibility sub-dimension were significantly higher for women. A review of the relevant literature shows that women generally have higher healthy lifestyle behaviors, similar to our study.^11-13^ Those who were not married had higher scores in these sub-dimensions. Although there are different sub-dimensions in the literature, in parallel with this study, it is generally seen that the unmarried group has more healthy lifestyle behaviors.^14-17^ Based on our study, it is thought that the effective factor in higher stress management and physical activity scores is that unmarried people have more time to spare for themselves, so they apply stress management techniques better and spend more time on physical activity.^18^

It is thought that those who do not have children apply stress management techniques better because they have fewer responsibilities and more time for themselves. In our study, physician income status did not significantly differ between groups on any of the general scale scores or sub-dimension scores. In the literature, healthy lifestyle behaviors of those with a good income status were found to be high.^11,15,19 20^ The difference between the results of the literature and the results of our study may be due to the difference in the groups in which the studies were conducted. In our study, the health responsibility sub-dimension score and the general scale score of non-smokers were found to be significantly higher. The fact that the general score of healthy lifestyle behaviors of non-smokers was found to be high in the literature supports our study.^13,15,21,22^

The fact that individuals who pay more attention to their health also avoid smoking is believed to be a situation that supports and overlaps with each other. In our study, the score for the health responsibility subscale among physicians with chronic diseases was found to be high.

Another factor thought to affect healthy lifestyle behaviors in our study was health perception. In our study, a significant difference was found only in interpersonal relationships and physical activity sub-dimension scores according to health perception.

In terms of our study, it is thought that the effective factor in the higher interpersonal relations scores of the participants with good health perception is that these people have better health and a healthy life is effective and important in establishing good relationships with people. In addition, the high physical activity scores of the participants with poor health perception in our study may have led them to engage in more physical activity because they perceived their health as poor.

In this study, being in a yountxger age group (23-28 years), not being married or having children, not smoking, and having a chronic disease were found to be determinants of healthy lifestyle behaviors, and the following recommendations were made:

- Opportunities for physical activity can be increased within or close to the institution where the physicians work.
- To increase the health responsibility scores of male participants, programs can be organized to encourage healthy lifestyles.
- The physicians should be encouraged to practice more stress management techniques and to engage in physical activity during the time left over from the responsibilities of marriage and children.
- Doctors who smoke can be referred to smoking cessation clinics, and they can be given priority in these clinics because they are role models for society.

It is also recommended that these factors be analyzed and studies carried out regarding modifiable characteristics for health promotion.

## Data Availability Statement

The data that support the findings of this study are available on request from the corresponding author.

## Figures and Tables

**Table 1.** Distribution of Sociodemographic Characteristics of Physicians

Feature	Number (n)	Percentage
Duty		
Research assistant	37 4	52.5
Final-year medical students	33 8	47.5
Age groups		
23-28	534	75.0
29-34	160	22.5
35 and above	18	2.5
Gender		
Woman	35 9	50.4
Male	35 3	49.6
Marital status		
Married	16 2	22.8
Unmarired	56 0	77.2
Having children		
There is	86	12.1
None	62 6	87.9
Income status		
Good	13 3	18.7
Moderate	45 4	63.7
Poor	12 5	17.6

The values in the table are presented as SD, mean, median, and interquartile distances.

**Table 2.** Distribution of Participants’ HPLP II Scores in Terms of Some Factors

		Overall Score	Nutrition	Physical Activity	Health Responsibility	Stress Management	Spiritual Development	Interpersonal Relationships
Gender	Woman	120.8 ± 15.4	18.8 ± 3.4	16.0 (7)	19.0 ± 3.9	18.1 ± 3.3	25.0 ± 4.2	24.7 ± 3.7
	Men	119.2 ± 17.5	18.9 ± 3.8	16.0 (6)	18.3 ± 3.8	17.6 ± 3.6	24.6 ± 4.7	23.6 ± 4.0
	Statistics	*P* = .193	*P* = .923	*P *= .093	*P* = **.010**	*P* = .108	*P* = .214	***P* < .001**
Age groups	23-28	120.7 ± 16.3	18.9 ± 3.6	16.1 ± 4.4	18.8 ± 3.9	18.1 ± 3.4^a^	24.8 ± 4.4	24.2 ± 3.8
	29-34	117.7 ± 17.0	18.6 ± 3.8	15.4 ± 4.3	18.3 ± 3.7	17.4 ± 3.5^a^	24.8 ± 4.5	24.0 ± 4.0
	35 and above	122.0 ± 18.0	19.5 ± 2.7	15.5 ± 4.5	19.09 ± 3.5	17.9 ± 3.3	24.4 ± 4.8	25.4 ± 4.8
	Statistics	*P* = .124	*P* = .468	*P* = .215	*P* = .333	*P* = **.007**	*P* = .941	*P* = .383
Marital status	Married	118.1 ± 17.2	18.5 ± 3.6	15.0 (6)	18.5 ± 3.8	17.2 ± 3.5	24.8 ± 4.7	24.1 ± 4.1
	Not married	120.6 ± 16.3	19.0 ± 3.6	16.0 (6)	18.7 ± 3.9	18.0 ± 3.4	24.8 ± 4.4	24.2 ± 3.8
	Statistics	*P* = .086	*P* = .176	*P* = **.049**	*P* = .532	*P* = **.012**	*P* = .864	*P* = .901
Child status	Having a child	118.3 ± 16.4	18.8 ± 3.7	14.0 (6)	18.5 ± 4.1	17.0 ± 3.5	25.1 ± 4.5	24.2 ± 4.2
	Not having a Child	120.3 ± 16.5	18.9 ± 3.6	16.0 (6)	18.7 ± 3.8	18.01 ± 3.5	24.7 ± 4.4	24.1 ± 3.9
	Statistics	*P* = .308	*P* = .862	*P* = .222	*P* = .631	*P* = **.020**	*P* = .577	*P* = .853
	Yes	117.1 ± 17.0^a^	18.3 ± 3.8	15.7 ± 4.1	18.0 ± 4.1^a^	17.5 ± 3.9	24.0 (6)	23.7 ± 3.8
Smoking	No	121.0 ± 16.2^a^	19.0 ± 3.5	16.0 ± 4.4	19.8 ± 3.7^a^	17.9 ± 3.3	25.0 (6)	24.3 ± 3.9
	Quit	117.0 ± 19.2	17.9 ± 3.5	15.9 ± 3.9	18.8 ± 5.5	17.8 ± 4.0	23 (6)	24.0 ± 4.8
	Statistics	*P* = .024	*P* = **.102**	*P* = .780	*P* = **.026**	*P* = .480	*P* = .527	*P* = .332
	Good	121.0 ± 16.5	19.1 ± 3.6	16.0 (6)^a^	19.0 (5)	18.0 ± 3.4	24.9 ± 4.4	24.4 ± 3.8^a^
Health perception	Moderate	118.7 ± 16.0	18.5 ± 3.5	15.0 (5)^b^	18.0 (4)	17.6 ± 3.5	24.7 ± 4.4	24.0 ± 3.9
	Poor	116.5 ± 21.3	18.5 ± 4.5	19 (8)^a,b^	18 (5)	17.1 ± 4.3	23.3 ± 4.6	21.7 ± 4.3^a^
	Statistics	*P* = .138	*P* = .077	*P* = **.007**	*P* = .416	*P* = .185	*P* = .245	*P* = **.006**

Significance values are highlighted in bold.

**Table 3.** Distribution of Scores of Assistant and Intern Physicians According to Sub-dimensions

	Assistant	Intern	Statistics
Physical activity	15.0 (6)	16.0 (7)	*P* < .001
Nutrition	18.7 ± 3.6	19.1 ± 3.6	*P* = .130
Health responsibility	18.5 ± 3.8	18.8 ± 3.9	*P* = .260
Stress management	17.4 ± 3.3	18.4 ± 3.5	*P* < **.001**
Spiritual development	24.8 ± 4.4	24.8 ± 4.5	*P* = .930
Interpersonal relationships	24.2 ± 3.8	24.1 ± 3.9	*P* = .530
Overall score	118.7 ± 16.0	121.6 ± 16.9	*P* = **.020**
